# A rare account of incidentally discovered anal melanoma

**DOI:** 10.1093/jscr/rjae728

**Published:** 2024-11-25

**Authors:** Nawal Khan, Dondre Irving, Lynn O’Connor

**Affiliations:** Department of Surgery, Wyckoff Heights Medical Center, Brooklyn, NY, United States; Department of Surgery, Wyckoff Heights Medical Center, Brooklyn, NY, United States; Department of Colorectal Surgery, Huntington Hospital, Huntington, NY, United States

**Keywords:** anal melanoma, anal cancer, treatment

## Abstract

Anal melanoma is a rare and highly aggressive malignancy that carries a poor prognosis. Due to its variable and ambiguous presentation, it is often misdiagnosed as a hemorrhoid, polyp, or an ulcer with a concomitant rectal prolapse. Clinicians usually have a low suspicion of anal melanoma due to its rarity and most people present with metastatic disease at the time of diagnosis. We report a case of a patient incidentally found to have anal melanoma. Prompt surgical resection with wide local excision versus abdominoperineal resection remains the mainstay of treatment as the added benefit of adjuvant chemoradiation or immunotherapy has been controversial.

## Introduction

Anal melanoma is a rare and highly aggressive malignancy, with an incidence ranging from 0.4% to 3.0% of all malignant melanomas and 0.1% to 4.6% of all anorectal malignant tumors [1]. It affects both men and women equally and most frequently occurs in the 5th and 6th decades of life [2, 3]. Often, it is misdiagnosed as a hemorrhoid, polyp, or ulcer, accompanied by rectal prolapse. Due to its variable and ambiguous presentation, diagnosis is challenging, leading to a poor prognosis. Here, we present a case of a 71-year-old female incidentally diagnosed with anal melanoma.

## Case report

A 71-year-old female with a medical history significant for Alzheimer’s disease, arthritis, bladder prolapse, hypertension, hyperlipidemia, pulmonary embolism, and varicose veins presented to the colorectal office due to bright red bleeding per rectum persisting for one and a half months, necessitating the use of pads. Additionally, she reported constipation and straining during bowel movements, accompanied by an unspecified amount of weight loss. Her last colonoscopy in 2021 yielded negative results, and there was no notable family history of colorectal cancer or polyps.

**Figure 1:**
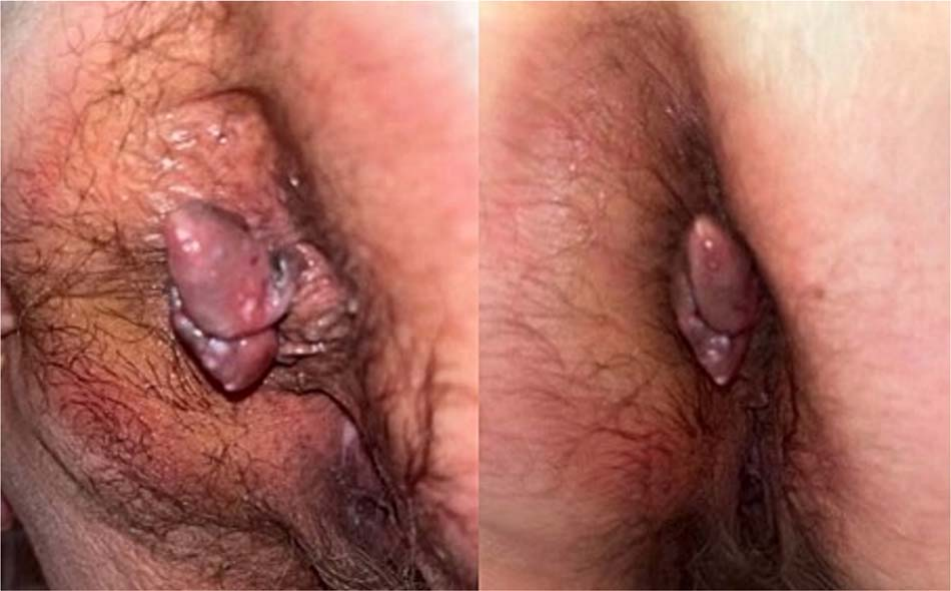
Images of anal melanoma on physical exam pre-surgical excision.

**Figure 2:**
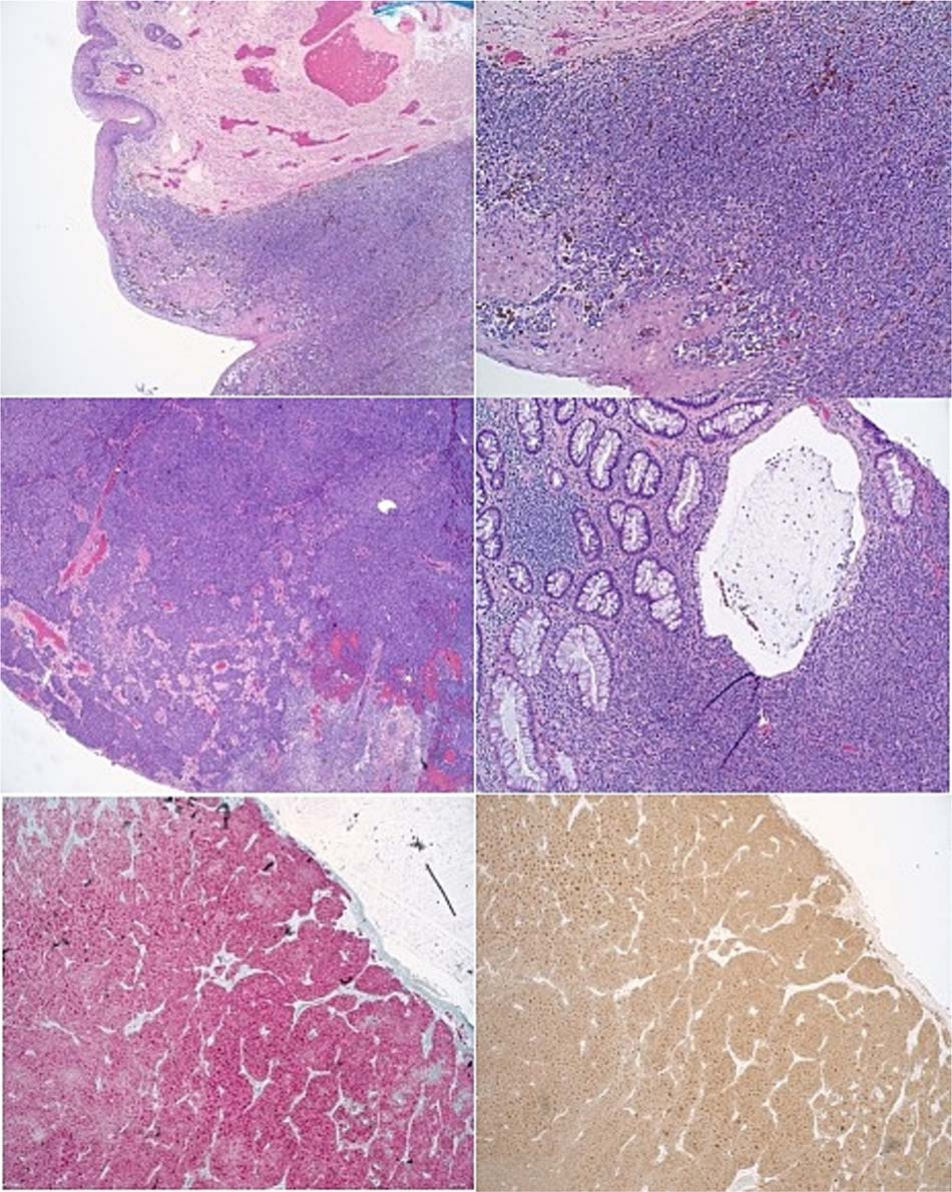
Histopathological slides of anal mass showing anal melanoma.

During the physical examination, a large 4-cm posterior midline bilobed friable mass was observed at the anal verge, exhibiting bleeding upon contact (Fig. 1). No preoperative laboratory investigations were conducted. Subsequently, the patient underwent rectal examination under anesthesia, revealing an ~4 cm mass at the anal verge characterized by friability and hardness with a thrombosed component. The mass was excised, and pathological analysis confirmed a diagnosis of malignant melanoma (see Fig. 2 for pathology images).

Following the operation, the patient underwent a staging CT scan, which revealed stable small clustered millimeter-sized nodules in the right middle lobe of the lung, consistent with findings from a previous examination six months prior. Additionally, the scan identified a diffusely heterogeneous enlarged thyroid and stable splenic hypodensities. Serpiginous clustered tubular densities along the inferior margin of the stomach, unchanged from imaging conducted five and a half years ago, were presumed to represent venous or lymphatic malformation. Subsequent colonoscopy findings were unremarkable. The patient is currently scheduled to commence immunotherapy.

## Discussion

Anal melanoma originates from melanocytic cells located in the dentate line and may manifest as either anal, rectal, or both. It typically spreads through the submucosal planes and drains into the inguinal and inferior mesenteric lymph nodes [2]. The tumor cells typically form nests and exhibit positive staining for S-100, HMB-45, and vimentin [3].

Diagnosing anal melanoma presents challenges due to its location and the delayed presentation of patients. Clinicians often have a low suspicion due to its rarity. Common presenting symptoms include bleeding (55% of cases), a perianal or rectal mass (34%), and pain (13%) [2]. Additionally, patients may experience pruritus, tenesmus, hemorrhoids, and changes in bowel habits. The disease progresses indolently, making diagnosis difficult, and most individuals have metastatic disease by the time of diagnosis, with the liver, lung, and brain being the most common sites of metastasis [2].

Diagnostic workup should encompass all possible differential diagnoses of a gastrointestinal mass or bleed. A high index of suspicion on physical examination should prompt a colonoscopy with tissue biopsy to confirm the diagnosis and identify synchronous lesions. MRI is the preferred imaging modality for delineating anorectal tissue planes and aiding in surgical planning.

Despite medical advancements, there is no consensus on the optimal treatment for anorectal melanoma due to the lack of clinical trials. While the roles of adjuvant chemotherapy, radiation, and immunotherapy remain controversial, surgical therapy is universally recommended. However, there is an ongoing debate regarding whether wide local excision or abdominoperineal resection (APR) is more appropriate for achieving complete cure. Although APR is a highly morbid procedure, it is theoretically superior as it limits lymphatic dissemination and provides a wider negative margin for local control [4]. However, a study in 1995 reported 64-year experience of surgeons at MSK with anorectal cancer and found that in patients with resectable disease, there was a higher disease free survival distribution of patients who underwent APR, although not statistically significant [5]. Recently, a systematic review and meta-analysis corroborated these findings and reported no significant survival benefit, irrespective of tumor stage, when the two surgical modalities were compared [6].

Since anorectal melanoma tends to recur early with aggressive metastases, with a median survival rate of 19–24 months and a 5-year survival rate of 10–17% [3, 5], the risks and benefits of surgical options must be carefully weighed in the context of the patient’s quality of life. It is suggested that patients with stages 0 and 1 disease undergo sphincter-sparing local excision with adjuvant radiation if needed to control local disease effectively while minimizing functional morbidity [3]. Patients with stage IV disease may benefit from APR to improve quality of life by alleviating symptoms of bleeding or tumor obstruction [7], although it does not affect the incidence of distant metastasis or survival.

Interestingly, the stage of melanoma at the time of diagnosis and tumor thickness have been found to be the most predictive factors of outcome, rather than the type of surgery performed [2]. Females also tend to have a more favorable prognosis.

## Conclusion

Anorectal melanoma is a rare malignancy that most often presents with regional or distant metastases at the time of diagnosis, making early detection crucial. No treatment modality, regardless, of how aggressive, has shown to improve survival benefit. Therefore, clinicians must maintain a high index of suspicion when encountering patients with symptoms such as anorectal bleeding, pain, or mass to facilitate early intervention.
